# Psychoactive substance use among medical residents in Tunisia

**DOI:** 10.1192/j.eurpsy.2022.939

**Published:** 2022-09-01

**Authors:** R. Masmoudi, I. Chaari, Y. Mejdoub, R. Sallemi, I. Feki, J. Masmoudi

**Affiliations:** 1 Hospital university of HEDI CHAKER, Psychiatry A Department, Sfax, Tunisia; 2 Hedi Chaker university hospital, Epidemiology, Sfax, Tunisia

**Keywords:** substance use, medical residents, Tunisia

## Abstract

**Introduction:**

Recent studies in the word found an increase of substance use among medical students.

**Objectives:**

To determine the prevalence of substance use and associated factors among medical residents in Tunisia.

**Methods:**

It was a descriptive and analytical cross-sectional study among medical residents from the 4 medical faculties of Tunisia. A questionnaire was created from Google Forms and was published on the social network Facebook. We asked about the current consumption of different psychoactive substances. We used the Patient Health Questionnaire (PHQ-9) to identify depressive symptoms.

**Results:**

The sample included 241 residents. The female sex was predominant (83.4%, n = 201). The average age was 28.18 (± 2.13) years. Among these residents, 27.8% (n = 67) currently consume at least one psychoactive substance and 71% (n = 171) had depressive symptoms. The substances consumed by residents were: tobacco 18.7% (n = 45), alcohol 18.7% (n = 45), cannabis 6.2% (n = 15) , amphetamine 3.3% (n = 8), sleeping pills (without medical prescription) 2.9% (n = 7), hallucinogens 2.9% (n = 7), cocaine 2.1 % (n = 5) and inhaled solvents 0.4% (n = 1).

The use of at least one psychoactive substance was significantly associated with male sex (p = 0.01), the presence of financial problems (p = 0.08), lack of religiosity (p <0.001), feeling of life dissatisfaction (p = 0.01), uncertainty about life events (p = 0.05) and the presence of depression (p = 0.018).

**Conclusions:**

Psychoactive substance use has become a growing problem among residents in Tunisia. The associated factors should attract attention to identify these subjects.

**Disclosure:**

No significant relationships.

